# Magnesium Citrate Capsules in Colonoscopy Preparation: A Randomized Controlled Trial

**DOI:** 10.7759/cureus.20506

**Published:** 2021-12-18

**Authors:** Yehuda Eidensohn, Yisroel Mond, Isaac Labowitz, Patricia Greenberg, Brielle Formanowski, Chaya Eidensohn, Sudhir Dutta, Ethan Dubin

**Affiliations:** 1 Internal Medicine, New Jersey Medical School, Newark, USA; 2 Gastroenterology and Hepatology, EndoCentre of Baltimore, Baltimore, USA; 3 Biostatistics and Epidemiology, Rutgers University, Piscataway, USA; 4 Gastroenterology and Hepatology, Sinai Hospital of Baltimore, Baltimore, USA; 5 Gastroenterology and Hepatology, University of Maryland School of Medicine, Baltimore, USA

**Keywords:** screening colonoscopy, colon cancer screening, colon cleanliness, magnesium citrate capsules, colonoscopy preparation

## Abstract

Introduction: Screening colonoscopies are recommended for the detection and prevention of colon cancer. Liquid colonoscopy preparations may be poorly tolerated. We evaluated the adequacy and tolerability of a novel low-cost colonoscopy preparation consisting of magnesium citrate capsules and bisacodyl (MCCB).

Methods: This is a single-center, assessor-blinded, randomized controlled trial of 51 patients undergoing screening colonoscopies, who received a bowel preparation of either 4 liters of GoLYTELY (Braintree Laboratories, Inc., Braintree, MA) or MCCB. The primary outcome was the rate of adequate colon cleanliness, defined as a total score ≥ 6 on the Boston Bowel Preparation Scale and no colon segment with a score of zero. The secondary outcome was patient satisfaction, assessed by a validated questionnaire.

Results: A total of 100% of patients in both arms achieved adequate colon cleanliness, and the magnesium citrate arm had superior patient satisfaction (mean satisfaction score: 54.8 vs. 172.8; p < 0.001).

Conclusions: A pill-based colonoscopy preparation of MCCB may be a low-cost option for patients reluctant to consume a liquid preparation.

## Introduction

Screening colonoscopies are recommended for the detection and prevention of colon cancer [1]. Patient adherence is suboptimal, and the preparation is an often-cited barrier to getting a colonoscopy [2]. The most commonly prescribed preparation is 4 liters of polyethylene glycol 3350 (PEG), which has low tolerability due to the taste and large volume. A pill-based preparation is potentially more tolerable to patients [3,4]. Several pill-based preparations have been approved by the Food and Drug Administration (FDA), including OsmoPrep (Salix, Bridgewater, NJ) and Sutab (Braintree Laboratories, Inc., Braintree, MA), but each cost over $100, which limits their widespread utilization [5]. Magnesium citrate liquid is a widely used preparation, and magnesium citrate capsules are commercially available as a supplement. We designed and evaluated a magnesium citrate capsule-based preparation, which costs approximately $3, with the hypothesis that it would have reasonable efficacy in colon cleanliness and superior patient satisfaction compared to 4 liters of GoLYTELY (Braintree Laboratories, Inc., Braintree, MA) preparation.

## Materials and methods

Recruitment and randomization

**Figure 1:**
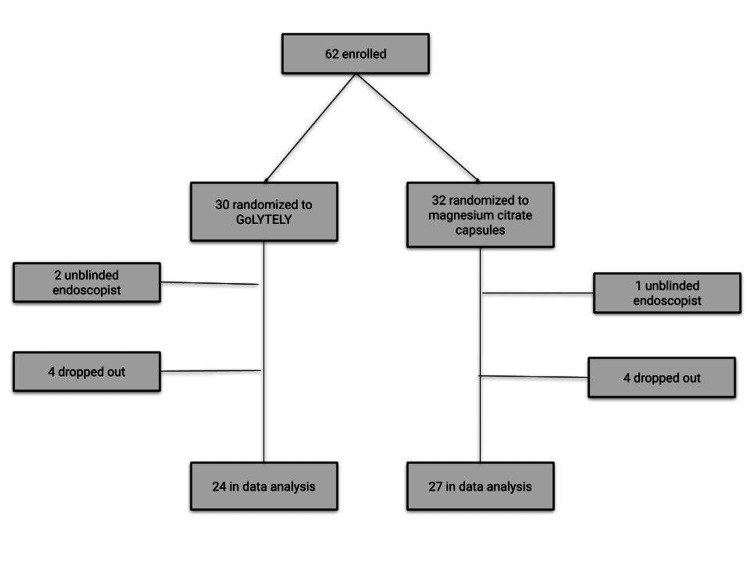
Participant flow in a randomized controlled trial of magnesium citrate capsules for colonoscopy preparation.

A single-center, assessor-blinded, randomized controlled trial was conducted from 2017 to 2020. Patients over 18 years referred for screening colonoscopies were screened for eligibility. Exclusion criteria were estimated glomerular filtration rate (eGFR) under 30 mL/min/1.73 m^2^, based on laboratory studies within the prior 30 days; taking proton pump inhibitors or H2 blockers and unable to stop for five or two days before the colonoscopy, respectively; achlorhydria; colon disease; previous colon surgery; cardiovascular disease; and decompensated cirrhosis with ascites. We excluded patients with decreased gastric acid secretion because capsule dissolution may rely on low gastric pH [6]. Eligible patients were randomized with computerized random numbers, in a 1:1 ratio, to receive either 4 liters of GoLYTELY or magnesium citrate capsules and bisacodyl (MCCB) (Figure 1). Treatment allocation was concealed using sequentially numbered sealed envelopes and staff involved in generating the sequence (YE) was not involved in the treatment assignment.

Preparation details

The pill-based preparation consisted of 36 Natural Factors® (Monroe, WA) 150 mg magnesium citrate capsules and four bisacodyl 5 mg tablets, which are commercially available without a prescription. Approved magnesium citrate preparations contain between 4.2 and 6.6 grams of magnesium [7], which is equivalent to 28 and 44 capsules, respectively. Pilot testing of magnesium citrate capsules showed that 36 capsules, containing 5.4 grams of magnesium, provided an optimal balance between efficacy and pill burden. The study was considered exempt from Investigational New Drug (IND) status since the active ingredient of magnesium citrate is already available in a liquid form. Subjects randomized to GoLYTELY were instructed to start approximately 15 hours before their colonoscopy, and drink 8 ounces every 10 minutes until 2 liters were consumed and the remaining 2 liters six hours before their colonoscopy in the same pattern. Subjects randomized to MCCB were instructed to start approximately 17 hours before their colonoscopy, and take three magnesium citrate capsules with 8 ounces of fluid every 15 minutes, until 18 capsules were consumed. The remaining 18 capsules were to be consumed approximately 12 hours before their colonoscopy in the same pattern. The bisacodyl tablets were to be taken at 16, 15, 11, and 8 hours before their colonoscopy.

Blinding

The endoscopist who assessed colon cleanliness was blinded to patient assignment through patient instruction to not disclose their preparation to the endoscopist. Patient blinding was not considered feasible.

Ethics approval

All subjects provided written informed consent. The study was approved by the LifeBridge Health Institutional Review Board (IRB) and registered prospectively on ClinicalTrials.gov (NCT03247595).

Data collection

The primary outcome was colon cleanliness, assessed using the Boston Bowel Preparation Scale, which was graded by an experienced endoscopist performing the colonoscopy [8]. An adequate preparation was defined prospectively as a total score ≥ 6, and no colon segment with a score of zero or one. The secondary outcome was tolerability and patient satisfaction, assessed by a validated questionnaire [9]. The questionnaire consists of six questions regarding the ability to consume the preparation, the experience and taste of the preparation, and willingness to take the preparation in the future. Each question was analyzed independently. Additionally, the first four questions were converted into a numeric score of 0-400, with 0 being the most tolerable.

Statistical analyses

Categorical data were analyzed using the chi-squared or Fisher’s exact test. Continuous data were analyzed using t-test or Wilcoxon rank-sum test, depending on the distribution of the variable. A two-sided p-value of <0.05 was considered significant. Data were used from per-protocol (PP) patients. For the purposes of this study, adequate bowel preparation was defined as a six or higher on the Boston Bowel Preparation Scale and no colon segment with a score of zero. The study was originally designed as a two-center non-inferiority study with a target sample size of 152, based on the power of 0.8 to detect a 7.5% difference in the proportion with adequate preparation, assuming a 20% dropout rate. However, the loss of the second center due to administrative challenges rendered the target sample size unattainable, and thus non-inferiority of colon cleanliness could not be demonstrated. Therefore, only descriptive statistics were used for the primary outcome. SAS version 9.4 (SAS Institute, Cary, NC) was used for analysis.

## Results

Enrollment

A total of 62 patients were enrolled, with 51 completing all study procedures. Enrollment details are provided in Figure 1. Subjects were 71% male, and the average age was 58 years, as described in Table 1. Treatment arms were comparable with respect to gender, race, BMI, prior colonoscopy, and diabetes. However, the MCCB arm was significantly older (mean age 60 vs. 55 in the GoLYTELY arm; p = 0.04).

**Table 1:** Characteristics of patients. ^a^ P-values from Wilcoxon rank-sum test. ^b^ P-values from Fisher’s exact test. ^c^ One subject in the liquid group did not report if they had a previous colonoscopy. ^d^ P-value from T-test.

Characteristic	Capsules (n = 27)	Liquid (n = 24)	p-value
Age (years), mean (SD)	60.0 (7.3)	55.1 (8.0)	0.04^a^
Race, n (%)			1.00^b^
White	14 (51.9)	13 (54.2)	
Black	12 (44.4)	11 (45.8)	
Not reported	1 (3.7)	0 (0.0)	
Male gender, n (%)	19 (70.4)	17 (70.8)	0.97^a^
BMI, mean (SD)	29.4 (4.3)	30.4 (7.7)	0.80^a^
Previous colonoscopy, n (%)	18 (66.7)	10 (43.5)^c^	0.15^d^
Diabetes	1 (3.7)	3 (12.5)	0.33^b^
Thyroid disease	3 (11.1)	2 (8.3)	1.00^b^

Colon cleanliness

Details of the Boston Bowel Preparation Scale are presented in Table 2. All patients in both arms had adequate preparation, with a total Boston Bowel Preparation Scale score ≥ 6 and no colon segment with a score of zero or one. The mean total score and standard deviation (SD) were 8.0 (1.0) in the capsule arm and 8.3 (0.7) in the GoLYTELY arm.

**Table 2:** Colon cleanliness as measured by the Boston Bowel Preparation Scale. ^a ^Each segment was scored as follows [8]: 0 = unprepared colon segment with mucosa not seen due to solid stool that cannot be cleared; 1 = portion of the mucosa of the colon segment seen, but other areas of the colon segment not well seen due to staining, residual stool, and/or opaque liquid; 2 = minor amount of residual staining, small fragments of stool and/or opaque liquid, but mucosa of colon segment seen well; and 3 = entire mucosa of colon segment seen well with no residual staining, small fragments of stool, or opaque liquid. ^b ^Only descriptive statistics are presented, as the sample size was not adequate to demonstrate non-inferiority. ^c^ Right colon including the cecum and ascending colon. ^d^ Transverse colon including the hepatic and splenic flexures. ^e ^Left colon including the descending colon, sigmoid colon, and rectum. ​​​​​​​^f ^Predefined as total score ≥ 6, and no colon segment with a score of zero or one.

Outcome^a,b^	Total (n = 51)	Capsules (n = 27)	Liquid (n = 24)
Right score^c^, mean (SD)	2.5 (0.5)	2.5 (0.5)	2.6 (0.5)
Right score, ordinal, n (%)			
2	23 (45.1)	14 (51.9)	9 (37.5)
3	28 (54.9)	13 (48.1)	15 (62.5)
Transverse score^d^, mean (SD)	2.9 (0.3)	2.8 (0.4)	3.0 (0.2)
Transverse score - ordinal, n (%)			
2	6 (11.8)	5 (18.5)	1 (4.2)
3	45 (88.2)	22 (81.5)	23 (95.8)
Left score^e^, mean (SD)	2.7 (0.5)	2.7 (0.5)	2.8 (0.4)
Left score, ordinal, n (%)			
2	15 (29.4)	9 (33.3)	6 (25)
3	36 (70.6)	18 (66.7)	18 (75)
Total score, mean (SD)	8.1 (0.9)	8.0 (1.0)	8.3 (0.7)
Total score, ordinal, n (%)			
6	4 (7.8)	3 (11.1)	1 (4.2)
7	5 (9.8)	5 (18.5)	0 (0)
8	22 (43.1)	9 (33.3)	13 (54.2)
9	20 (39.2)	10 (37)	10 (41.7)
Adequate^f^ colon cleanliness, n (%)	51 (100)	27 (100)	24 (100)

Tolerability

Tolerability was superior in the magnesium citrate capsule arm, with 63% of patients reporting the preparation being “very easy” to consume vs. 9% in the GoLYTELY arm (p < 0.001) (Table 3). Of patients in the capsule arm, 96% reported being able to consume the entire preparation, vs. 74% in the GoLYTELY arm (p = 0.04). The taste of the preparation was reported as “excellent” or “good” by 82% of the capsule arm vs. 13% of the GoLYTELY arm (p < 0.001). A total of 82% of the capsule arm would ask for the same preparation in the future vs. 35% of the GoLYTELY arm (p = 0.001). The results were similar when patients were stratified by previous colonoscopy experience. No serious adverse events were reported in either arm.

**Table 3:** Tolerability. ^a^ One subject in the liquid group did not complete the questionnaire. ^b^ P-values from Wilcoxon rank-sum test. ^c^ P-values from Fisher’s exact test. ^d ^P-value from the chi-square test. ^e ^Q1, Q3, and Q4 are scored 0-4 with 0 being most tolerable; then multiplied by 25. Q2 is scored 0 for yes and 1 for no; then multiplied by 100. The two numbers are summed for a total score. A total score of 0 is the most tolerable score, and 400 is the least tolerable score.

Outcome	Total (n = 50^a^)	Capsules (n = 27)	Liquid (n = 23^a^)	p-value
Q1: How easy or difficult was it to consume the study drug? n (%)				<0.001^b^
Very easy	19 (38.0)	17 (63.0)	2 (8.7)	
Easy	9 (18.0)	5 (18.5)	4 (17.4)	
Tolerable	15 (30.0)	4 (14.8)	11 (47.8)	
Difficult	6 (12.0)	1 (3.7)	5 (21.7)	
Very difficult	1 (2.0)	0 (0.0)	1 (4.3)	
Q2: Able to consume the entire preparation as instructed. n (%)	43 (86.0)	26 (96.3)	17 (73.9)	0.04^c^
Q3: Describe your overall experience of the study preparation: n (%)				0.006^b^
Excellent	15 (30.0)	13 (48.1)	2 (8.7)	
Good	17 (34.0)	8 (29.6)	9 (39.1)	
Fair	13 (26.0)	4 (14.8)	9 (39.1)	
Poor	2 (4.0)	1 (3.7)	1 (4.3)	
Bad	3 (6.0)	1 (3.7)	2 (8.7)	
Q4: The taste of this study preparation was: n (%)				<0.001^b^
Excellent	17 (34.0)	16 (59.3)	1 (4.3)	
Good	8 (16.0)	6 (22.2)	2 (8.7)	
Tolerable	18 (36.0)	4 (14.8)	14 (60.9)	
Poor	4 (8.0)	0 (0.0)	4 (17.4)	
Bad	2 (4.0)	0 (0.0)	2 (8.7)	
Q5: Would ask for this preparation for a future colonoscopy. n (%)	30 (60.0)	22 (81.5)	8 (34.8)	0.001^d^
Q6: Would refuse the same preparation if prescribed in the future. n (%)	11 (22.0)	4 (14.8)	7 (30.4)	0.30^c^
Numeric score (based on Q1-Q4)^e^				<0.001^b^
Mean (SD)	110.2 (98)	54.8 (62.9)	172.8 (93.5)	
Median (Q1, Q3)	100 (25, 150)	37.5 (0, 100)	150 (125, 250)	

## Discussion

Screening colonoscopies play an essential role in the prevention and early detection of colon cancer. Adherence to screening guidelines is suboptimal. One cause of patient hesitation is the colonoscopy preparation, which has traditionally been a large volume of unpalatable liquid, such as PEG solution. This small randomized controlled trial demonstrated that a novel low-cost colonoscopy preparation of MCCB produced adequate colon cleanliness in 100% of patients, and was more tolerable to patients than 4 liters of GoLYTELY.

To our knowledge, this is the first reported study of magnesium citrate capsules for colonoscopy preparation. Sodium picosulfate-based preparations, which consist of sodium picosulfate, magnesium oxide, and anhydrous citric acid, form magnesium citrate in solution and have been evaluated in over 25 randomized controlled trials. A recent meta-analysis [10] reported non-inferiority of the colon cleanliness of sodium picosulfate-based preparations, compared to PEG, with a non-inferiority margin of 10%. Products based on sodium picosulfate approved by the FDA include Prepopik (Ferring, Saint-Prex, Switzerland), which has since been discontinued, and Clenpiq (Ferring, Saint-Prex, Switzerland) [11]. Patients found these low-volume preparations more tolerable than PEG-based preparations, although electrolyte abnormalities did occur in patients with kidney dysfunction [12]. Additionally, the wholesale acquisition price of Clenpiq is $153.50, which may limit widespread usage [11].

Pill-based colonoscopy preparations include OsmoPrep and Sutab. OsmoPrep consists of 32 sodium phosphate tablets and has demonstrated non-inferior colon cleanliness compared to PEG, as well as greater tolerability [4]. However, cases of acute phosphate nephropathy have been reported with sodium phosphate preparations, leading to a black box warning that may deter some clinicians from prescribing them. Sutab consists of 24 tablets of sodium sulfate, magnesium sulfate, and potassium chloride, and has demonstrated non-inferior colon cleanliness compared to both low-volume PEG and sodium picosulfate-based preparations [5]. The wholesale acquisition cost of OsmoPrep and Sutab are $247 and $150, respectively. This study preparation consisted of 36 magnesium citrate capsules and four bisacodyl tablets, with a cost of approximately $3.

Strengths of this study include its randomized design and assessor blinding. Limitations of the study include its small size, which does not allow for comparisons of colon cleanliness between groups. The colon cleanliness of the MCCB group should be regarded as a proof of concept only, rather than a demonstration of non-inferiority. Additionally, information was not collected on the total number of patients screened, which has the potential of selection bias. Third, information was not collected on specific patient-reported adverse effects, such as nausea, bloating, and abdominal pain. Fourth, although magnesium citrate preparations have an increased risk of electrolyte abnormalities compared to polyethylene glycol [11], follow-up laboratory studies were not routinely performed. However, all patients were required to have a baseline metabolic panel within the prior 30 days, and patients with significant renal dysfunction were excluded.

## Conclusions

In this small randomized controlled trial, a novel low-cost colonoscopy preparation of magnesium citrate capsules and bisacodyl produced adequate colon cleanliness in 100% of patients. It also demonstrated superior patient satisfaction and tolerability compared to 4 liters of GoLYTELY. Patients reluctant to consume a liquid colonoscopy preparation may be more willing to consider a pill-based preparation. The cost of this preparation is approximately $3, which may make it more accessible to patients than existing pill-based preparations. Further research is needed to evaluate the non-inferiority of colon cleanliness produced by magnesium citrate capsules compared to other preparations.
